# Recirculation Differences in Veno-Venous Extracorporeal Membrane Oxygenation By Different Types of Cannula Designs

**DOI:** 10.1186/2197-425X-3-S1-A504

**Published:** 2015-10-01

**Authors:** O Palmér, J Hultman, LM Broman

**Affiliations:** Karolinska University Hospital, Solna, ECMO Center Karolinska, Stockholm, Sweden

## Introduction

Effectiveness of veno-venous (VV) ECMO treatment is in part dependent on the magnitude of recirculation. the recirculation fraction (RF) is for example influenced by cannulation type (dual lumen or single lumen cannulas), cannula position, pump flow, and cardiac output. RF might also be affected by cannula design. Knowledge of RF related to a certain cannula design might influence the choice of cannula and hence improve treatment efficiency.

## Objectives

The purpose of this study was to examine the influence of ECMO flow on RF when using two different cannula designs for drainage of venous blood.

## Methods

All patients were submitted to peripheral cannulation, and atrio-femoral flow direction was used. the ELSA^TM^ (Transonic System Inc., Ithaca, USA) was used for measurement of recirculation during VV ECMO. Two ultrasound flow dilution sensors were clamped on the arterial and venous lines during VV ECMO in adults. Isotonic saline (15 to 20 ml) was injected before the oxygenator. ELSA displayed RF (%) of total ECMO flow. in all patients ECMO flow was varied and RF was measured. Two cannula designs were used; a multi-stage and one of conventional design. Statistics were performed with SPSS (Version 22.0, Armonk, NY, IBM Corp., USA). Linear regression, and correlation according to Pearson was performed for each cannula. Mann-Whitney U-test was used to compare the two different cannulas. a p-value < 0.05 was considered significant.

## Results

Between August 2014 and March 2015 10 adult patients (74.2 ± 11.4 (SD) kg, median 80 kg; 173 ± 16.1, median 177 cm) with respiratory failure were investigated. Time of measurement varied from day one to 19; 43 measurements were performed. in one patient both cannula designs were used.

**Figure 1:**
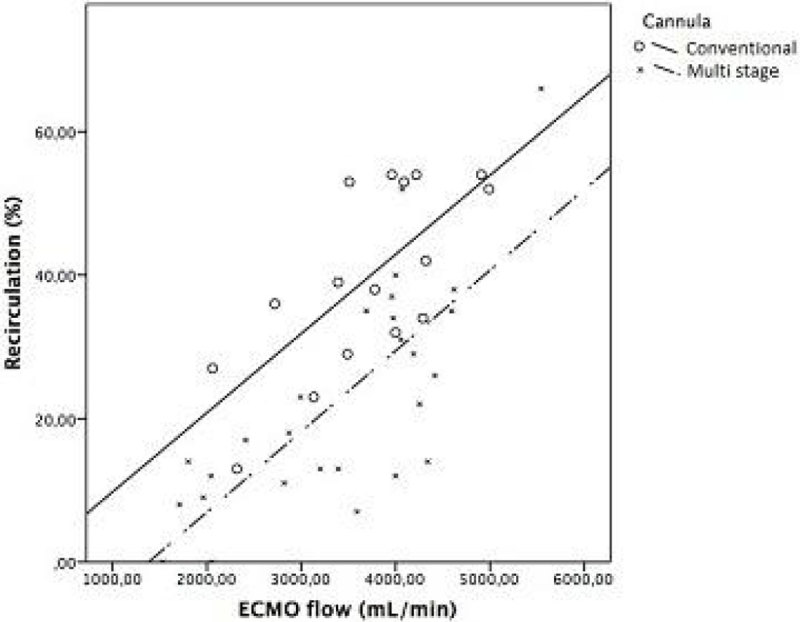
Figure 1

**Figure**1 shows the scatter plot of the recirculation fraction (RF, %) against the veno-venous ECMO flow, and the corresponding regression curve for each cannula, respectively. (Conventional cannula: R = 0.715, p = 0.002, n = 16; Multi stage cannula: R = 0.761, p = 0.0001, n = 27).RF was significantly lower for the staged cannula compared to the conventional one, p < 0.001.

## Conclusions

This study indicates less recirculation when using a multistage design draining cannula placed via the internal jugular vein with the tip mid atrial. Thus, just by the choice of draining cannula the patient can be offered a more efficient ECMO treatment. Continued studies are necessary for further evaluation.

